# A Prospective Evaluation of Two Rapid Phenotypical Antimicrobial Susceptibility Technologies for the Diagnostic Stewardship of Sepsis

**DOI:** 10.1155/2018/6976923

**Published:** 2018-05-10

**Authors:** Cesira Giordano, Elena Piccoli, Veronica Brucculeri, Simona Barnini

**Affiliations:** SD Ospedaliera di Microbiologia, Azienda Ospedaliero-Universitaria Pisana, Via Paradisa, No. 2, Ed. 200, 56124 Pisa, Italy

## Abstract

Rapid identification of bloodstream pathogens by MALDI-TOF MS and the recently introduced rapid antimicrobial susceptibility testing (rAST) directly from positive blood cultures allow clinicians to promptly achieve a targeted therapy, especially for multidrug resistant microorganisms. In the present study, we propose a comparison between phenotypical rASTs performed in light-scattering technology (Alfred 60AST, Alifax®) and fluorescence* in situ* hybridization (Pheno™, Accelerate) directly from positive blood cultures, providing results in 4–7 hours. Blood samples from 67 patients admitted to the Azienda Ospedaliero-Universitaria Pisana were analyzed. After the direct MALDI-TOF MS identification, the rAST was performed at the same time both on Alfred 60AST and Pheno. Alfred 60AST provided qualitative results, interpreted in terms of clinical categories (SIR). Pheno provided identification and MIC values for each antibiotic tested. Results were compared to the broth microdilution assay (SensiTitre™, Thermo Fisher Scientific), according to EUCAST rules. Using Alfred 60AST, an agreement was reached, 91.1% for Gram-negative and 95.7% for Gram-positive bacteria, while using Pheno, the agreement was 90.6% for Gram-negative and 100% for Gram-positive bacteria. Both methods provided reliable results; Alfred 60AST combined with MALDI-TOF MS proved itself faster and cheaper. Pheno provided identification and MIC determination in a single test and, although more expensive, may be useful whenever MIC value is necessary and where MALDI-TOF MS is not present.

## 1. Introduction

Sepsis is defined as life-threatening organ dysfunction caused by a dysregulated host response to infection and is the leading cause of death in intensive care units [1, 2]. It can cause long-term disability, prolonged hospitalizations, large additional costs for healthcare systems, and loss of quality of life for patients and their families [3]. Empirical antibiotic therapy administered within the first hour of clinical suspicion of sepsis decreases mortality rate but may have multiple negative effects related either to drug side effects or to the rise of multidrug resistance pathogens [4, 5]. Bacterial multidrug resistance is indeed emerging worldwide at an alarming rate and is recognized now as a major public health threat [6]. It is clear that the actual condition will not be resolved by the development of new antibiotics, as only few molecules have been recently introduced for humans [7, 8]. Therefore, in order to reduce selective pressure on microorganisms and effectively cure septic patients, an antimicrobial susceptibility profile is required as soon as possible. This will allow us to properly address the therapy, avoiding unnecessary antibiotic administrations. Thus, implementing strategies to preserve the activity of existing antimicrobial agents has become a healthcare priority, leading to the development of several antimicrobial stewardship programs [4, 5].

Several diagnostic systems have been developed for rapid identification of microorganisms found in positive blood cultures, providing quicker results than conventional methods [9]. In routine clinical practice, laboratory identification (ID) and antimicrobial susceptibility testing (AST) are based primarily on bacterial cultures and are usually completed in 2 days or more, during which time empirical therapy is initiated, based on the suspected causative organism and local epidemiology [10]. The use of new technologies able to rapidly detect antimicrobial resistance in bacterial isolates therefore has the potential to reduce the duration of empirical treatment and facilitate the early initiation of targeted therapy. Conventional AST systems indirectly sense change in bacterial population by measuring optical density and require 8–20 hours to obtain results, depending on the microorganism [11]. To date, various methods have been developed to improve conventional AST systems, the so-called rAST [12–17].

In this study, we provide a prospective evaluation of two phenotypical rASTs during sepsis: the Accelerate Pheno (Accelerate Diagnostics, USA) and Alfred 60AST (Alifax SpA, Italy) systems, compared to conventional culture-based identification and broth microdilution assay AST. Pheno is a fully automated system capable of performing identification and AST directly from positive blood cultures within approximately 7 hours. The system relies on gel electrofiltration and fluorescence in situ hybridization for bacterial identification, as well as automated digital microscopy for analyzing bacterial growth rates and for extrapolating MIC values. The Alfred 60AST system is based on a light-scattering technique that reliably detects microbial growth in fluid samples, providing real-time growth curves and bacterial counts (CFU/ml). The instrument allows only for detection of live bacteria, because the initial blank value reading eliminates inert materials. The system was previously evaluated for microorganism enrichment, determination of microbial count, and diagnosis of central venous catheter-related bloodstream infections [18, 19]. Alfred 60AST was initially conceived for urine screening and for AST of bacterial isolates from urine [20]. Then, it was adapted to rapid AST from positive monomicrobial blood cultures, coupled with a rapid direct identification using MALDI-TOF [21, 22]. AST results are available in 4–6 hours: in the presence of a specific drug, absence of growth is interpreted as sensitivity and growth as resistance to the antibiotic, in terms of clinical categories.

## 2. Materials and Methods

### 2.1. Blood Samples

Blood samples from 67 patients admitted to the Azienda Ospedaliero-Universitaria Pisana (Pisa, Italy) in the March-August 2017 period were inoculated into blood culture (BC) bottles (Becton, Dickinson & Co., Milan, Italy), collected at the SD Ospedaliera di Microbiologia and transferred to the Bactec FX instrument (Becton Dickinson) for monitoring bacterial growth. For each patient, two inclusion criteria were followed: (1) we chose the first positive BC, which was apparently monomicrobial at the Gram staining; (2) for Gram-positive cocci seen in grape-like clusters, we chose only BCs that had a time to positivity under 10 hours. Blood cultures containing Gram-negative bacilli from 55 patients and Gram-positive cocci from 12 patients were investigated. After subculture on blood agar plates (bioMérieux, Marcy l'Étoile, France), 5 of 55 (9%) and 2 of 12 (17%) cultures were found to be polymicrobial, and ID results were analyzed separately (see Section 3.3). Samples were taken as remnants of standard patient care and used anonymously. For this type of study, no written informed consent was necessary. Microbiology laboratory operation time is weekdays from 8 a.m. to 8 p.m. and weekends from 8 a.m. to 2 p.m. Blood cultures are processed every day during operating hours as soon as they are flagged positive.

### 2.2. Identification and AST of Bacteria by the Routine Method

From BCs found positive with the Bactec FX instrument (Becton Dickinson), Gram-staining and subcultures onto appropriate solid media—nutrients (e.g., blood agar) and selective plates (MacConkey and Mannitol Salt agar)—were performed, and then plates were incubated overnight at 37°C. Isolated colonies were identified using MALDI-TOF MS (Bruker Daltonics) and AST was performed with the SensiTitre system (Thermo Fisher Scientific, MA, USA), according to the manufacturer's instructions. Two different SensiTitre plates were used: ITGNEGF for Gram-negatives and ITGPOSF for Gram-positives. Colonies were dissolved in sterile water and the suspension was adjusted to 0.5 McFarland standard, using the Densimat densitometer (bioMérieux). For* Staphylococcus aureus* and* Enterococcus* spp. 50 *μ*L of the suspension was transferred into 11 mL Cation-Adjusted Mueller-Hinton Broth (CAMHB) (Thermo Fisher Scientific) and 100 *μ*L was inoculated into each well on the SensiTitre plate. For Gram-negatives 10 *μ*L was transferred into 11 mL CAMHB and 50 *μ*L of the suspension was inoculated into SensiTitre plate. Plates were incubated for 20–24 hours at 35°C in the ARIS™ Instrument (Thermo Fisher Scientific). The ID and AST results by the routine method were used as reference for evaluation of results obtained by MALDI-TOF MS and Pheno rapid ID, and Alfred 60AST and Pheno preliminary ASTs. Occasionally, the MIC test strip (Liofilchem, Roseto degli Abruzzi, Italy) was performed in case of ambiguous results.

### 2.3. Rapid Identification of Bacteria from Positive Blood Cultures

For the rapid identification method, herein referred to as direct ID according to a previous paper from Barnini and colleagues [22], a modified protocol was used. Briefly, an 8 ml sample of a positive BC at the Bactec FX was transferred into Serum Separator Tubes (BD Vacutainer systems). Next, bacteria were stratified on the surface of the silicon layer by centrifugation at 3500 rpm for 10 min. The bacterial pellet was entirely collected and suspended in 1 ml of distilled water into an eppendorf tube. Two centrifugations at 13,000 rpm for 2 min, alternated by adding 1 ml of distilled water, were performed, and then the bacterial pellet was allowed to dry at room temperature. If necessary, a further washing step was added in order to eliminate interfering erythrocytes. Next, bacteria were transferred with a wooden stick onto the polished steel target plate for MALDI-TOF MS identification. Gram-positive cocci were further exposed to a protein extraction protocol. In detail, Gram-positive cocci were overlaid with 1 *μ*l of absolute ethanol (Fluka, St. Louis, MO, USA). When dry, 1 *μ*l of formic acid (70% v/v; Fluka) was added and, when air-dried, 1 *μ*l of acetonitrile (Carlo Erba, Milan, Italy) was added. The preparation was overlaid with 1 *μ*l of saturated alpha-cyano-4-hydroxycinnamic acid (HCCA) in 50% acetonitrile and 2.5% trifluoroacetic acid matrix solution (Bruker Daltonics) and air-dried, thus allowing the crystallization of HCCA with the sample. MALDI-TOF MS analysis was performed using a Microflex LT system table top mass spectrometer following the manufacturer's instructions. Captured spectra were analyzed as reported elsewhere [22].

### 2.4. Rapid AST of Bacteria Using Pheno

Two Pheno system modules were available during our study period, allowing us to test two samples simultaneously. Quality controls were performed in advance, according to the manufacturer's instructions. As soon as the BC bottles were flagged positive, the Gram staining and the direct identification by MALDI-TOF MS were performed. Then, the Pheno Test BC kits (Accelerate Diagnostics) were run on the Pheno system modules. According to the company, it is recommended to apply the Pheno system within 8 hours after the BC is flagged positive. However, due to our working hours, we extended this period and included samples positive within 16 hours. One ml of the positive BC was transferred into the sample vial (Accelerate Diagnostics). The test system was then started, lasting approximately 5 min. Blood culture aliquots (500 *μ*l) were also stored at −80°C in order to evaluate eventual discrepancies. The analysis software, Accelerate Diagnostics Host applications, version 1.2.0.87, automatically generated first an ID report and subsequently an AST report. Pheno identified the following microorganisms:* Escherichia coli, Klebsiella *spp*., Enterobacter *spp*., Proteus *spp*., Citrobacter *spp*., Serratia marcescens, Acinetobacter baumannii, *and* Pseudomonas aeruginosa *among Gram-negatives and* Staphylococcus aureus*, Coagulase-Negative Staphylococci (CoNS),* Staphylococcus lugdunensis, Enterococcus faecalis, Enterococcus faecium, Streptococcus *spp.,* Streptococcus pneumoniae, *and* Streptococcus agalactiae,* among Gram-positives. AST was performed as reported in Tables 1(a) and 1(b).

### 2.5. Rapid AST of Bacteria Using Alfred 60AST

Direct AST of bacteria by Alfred 60AST (Alifax) was carried out by transferring 10 *μ*l of broth from positive BC into a vial containing 3 ml of HB&L enrichment broth (Alifax). Vials were loaded in the thermostatic area of the Alfred 60AST system, for monitoring bacterial growth up to 0.5 McFarland. Next, the instrument automatically transferred the sample into an AST-Empty vial (Alifax), placed in the refrigerated area. Lyophilized antibiotics (Alifax) were dissolved in 2 ml regenerating solution (provided with the antibiotics) and stored at 4°C for up to 6 days (3 days for meropenem). Regenerated antibiotics were loaded into the refrigerated area. The antibiotics tested were CE approved. Four panels were established, based on the antibiotics available, named as follows:* Enterobacteriaceae*, Gram-negative nonfermenters,* S. aureus*,* Enterococcus*.* Enterobacteriaceae* panel included 10 antibiotics: ceftazidime, cefotaxime, ceftriaxone, cefuroxime, piperacillin/tazobactam, meropenem, gentamicin, levofloxacin, colistin, and trimethoprim-sulfamethoxazole. Gram-negative nonfermenters panel included ceftazidime, amikacin, levofloxacin, gentamicin, and colistin. The* S. aureus* panel comprised cefoxitin, linezolid, teicoplanin, vancomycin, daptomycin, and clindamycin. The* Enterococcus* panel comprised ampicillin, linezolid, teicoplanin, vancomycin, and daptomycin. The vials dedicated to the AST analysis, each containing 2 ml of HB&L broth, were stored in the thermostatic area at 37°C and automatically loaded with the bacterial suspension (100 *μ*l), until 0.5 McFarland was reached, and with the selected antibiotic (200 *μ*l). One reference vial was loaded with 100 *μ*l of bacterial suspension only and used as a positive control for bacterial growth. Three dedicated algorithms, based on fast growing bacteria (e.g.,* Klebsiella pneumoniae*), medium-slow growing bacteria (e.g.,* Staphylococcus aureus*), and slow growing bacteria (e.g.,* Acinetobacter baumannii*), automatically calculated bacterial growth in the presence of each antibiotic, comparing the growth rate with the reference vial. The majority of the results were available within 3 hours, except for meropenem, piperacillin/tazobactam, teicoplanin, and vancomycin, for which 5 hours were required. Absence of growth was interpreted as sensitivity and growth as resistance to the antibiotic, in terms of clinical categories.

### 2.6. Data Analysis

The Pheno system identification results were compared with results from the direct identification and from culture-based identification, as routine procedure. The IDs were considered correct if the microorganism produced the same result as in the routine procedure, with no additional microorganisms detected. Polymicrobial cultures were, therefore, excluded from the analysis. The two rapid AST results were compared to the SensiTitre AST, interpreted according to EUCAST guidelines. The categorical agreement (CA) was determined for both methodologies, and the discrepant results were branded according to the current ISO 20776-2 guidelines as follows: very major errors (false susceptibility), major errors (false resistance), or minor errors (intermediate versus resistant or susceptible). The rate of very major errors (VMEs) was calculated dividing the number of false susceptible by the number of resistant strains tested and multiplied by 100, while the rate of major errors (MEs) was calculated dividing the number of false resistance by the number of susceptible strains tested and multiplied by 100. The rate of minor errors (MiEs) was calculated dividing the number of minor discrepancies by the total number of strains tested and multiplied by 100. The essential agreement (EA) that is the proportion of total test results within one doubling dilution of the reference result was calculated solely for Pheno system, since Alfred 60AST does not provide MIC values. Statistical analysis was performed using the paired sample *t*-test. The level of significance was set at *p* < 0.05.

## 3. Results and Discussion

### 3.1. The Hospital

**Figure 1:**
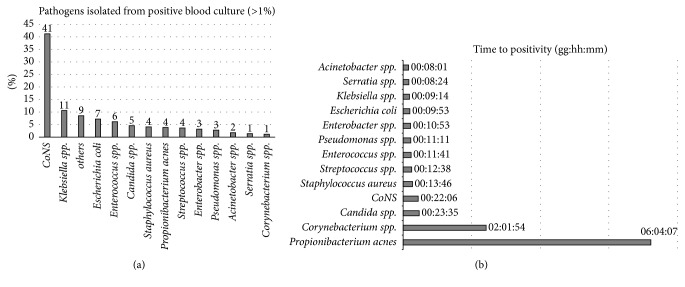
The 13 most frequently isolated microorganisms (>1%) from positive blood cultures in our laboratory during January-August 2017 (a). Median time to positivity of microorganisms in blood cultures (b).

The Azienda Ospedaliero-Universitaria Pisana is a tertiary-care university hospital, owning 1.300 beds and accounting for about 50.000 hospitalized patients each year. In 2017 (January-August), the microbiology laboratory processed 3630 positive blood cultures, which represented almost 14% of total BCs arrived. Of these, 95% were positive for bacteria and 5% for fungi. As showed in Figure 1(a), among Gram-positives, CoNS represented 41% of bacterial isolates, followed by* Enterococcus *spp. (6%) and* S. aureus* (5%). Common contaminants isolated included* Propionibacterium* spp. (4%),* Corynebacterium* spp. (1%), and* Micrococcus* spp. (<1%). Among Gram-negatives,* K. pneumoniae* was the principal microorganism isolated (11%), followed by* E. coli* (7%). For each patient, the first apparently monomicrobial BC positive for Gram-negatives was included in the study. Since CoNS are the main microorganisms isolated among Gram-positives but often represent a contamination in positive BCs, in order to exclude these microorganisms a further criterion of selection was adopted: the time to positivity of BCs. The median time to positivity (Figure 1(b)) for CoNS was 22 hours, while for* S. aureus* it was appreciably lower (13 hours); for this reason, for Gram-positive cocci in grape-like clusters, only BCs positive within 10 hours were included in the study. Several studies have been considering the prognostic value of time to positivity of BCs, as well as the cogency of this value for diagnosing catheter-related bloodstream infections [23–25]. We observed this trend since 2015 [26, 27] and, in our routine practice, we consider time to positivity as a predictor for* S. aureus* BSI.

### 3.2. Microorganisms Included in the Study

The performances of Pheno and Alfred 60AST systems were evaluated on a total of 67 positive BCs (Table 2); of these, 60 were monomicrobial and 7 polymicrobial. The following Gram-negative and Gram-positive monomicrobial infections were included in the comparative analysis: 50 caused by Gram-negatives, specifically* Acinetobacter baumannii* (*n* = 1),* Citrobacter koseri* (*n* = 3),* Enterobacter cloacae* (*n* = 4),* Escherichia coli* (*n* = 19),* Klebsiella pneumoniae* (*n* = 19),* Proteus mirabilis* (*n* = 1),* Pseudomonas aeruginosa* (*n* = 1), and* Serratia marcescens* (*n* = 2), and 10 caused by Gram-positives, precisely* Enterococcus faecalis* (*n* = 1),* Enterococcus faecium* (*n* = 3),* Staphylococcus aureus* (*n* = 4),* Streptococcus pneumoniae* (*n* = 1), and* Streptococcus agalactiae* (*n* = 1). Antimicrobial susceptibility results were produced for 74 isolates, with a total of 784 antimicrobial test results, 76 for Gram-positives and 708 for Gram-negatives. Five (42%) of the Gram-positive and 42 (79%) of the Gram-negative isolates were resistant to one or more antimicrobials. Interestingly, among* Klebsiella pneumoniae* strains, 10 (48%) were carbapenemase-producers, 9 of these codified for *bla*_KPC_ and one for *bla*_VIM_ genes, identified by the off-label use of the Xpert® Carba-R test (Cepheid, Sunnyvale, CA, United States) [28]. Four KPC-*Klebsiella pneumoniae* isolates were resistant to colistin, too. Six* E. coli* strains (32%) were resistant to one or more third-generation cephalosporins, and one was resistant to colistin. Among Gram-positives, one* E. faecium* isolate (33%) codified for* vanA* gene and one* S. aureus* (25%) was a methicillin-resistant strain.

### 3.3. Polymicrobial Blood Cultures

Among 67 BCs, 7 (10%) appeared to be polymicrobial when subcultured on blood agar plates. Most likely the proportion of the second microorganism was very low, since direct identification by MALDI-TOF MS detected only the first one, with a high score (Table 3). Pheno failed the ID and the AST for four polymicrobial BCs* (Enterococcus/Pseudomonas; Enterococcus/Streptococcus; Enterobacter/Staphylococcus; Klebsiella/Bacillus)*; for the couple* Klebsiella/Staphylococcus* it identified and provided the AST only for the Gram-negative, for the couple* Klebsiella/Enterococcus *it identified both the microorganisms and provided the AST for the* Enterococcus *spp.; finally, for the couple* Klebsiella/Citrobacter* it identified only* Klebsiella *spp. without providing the AST.

### 3.4. Identification of BSI Pathogens

Correct rapid identification by MALDI-TOF MS was achieved in all the monomicrobial BCs, although for two* Enterobacter cloacae* and one* S. aureus* infections the identification score was low (Table 2). To assess the reproducibility of the identification, the bacteria were spotted in duplicate and the results revealed 100% concordance and similar scores. Pheno was not able to provide results for 7 monomicrobial cultures, for 3 of these probably because the time elapsed from positivity to the BC processing was close to 16 hours, thus outside company recommendations. For 4 BCs (7%), the control growth failed without other explanations. These results show that Pheno processed 88% of BCs introduced in the instrument. Furthermore, Pheno identified an additional microorganism in 2 infections classified as monomicrobial by routine culture method. Particularly, a strain of* Serratia marcescens* was identified in a BC positive on plate as* Enterobacter cloacae* and a CoNS in a BC positive on plate as* Enterococcus faecium*. Therefore, the proportion of corrected identified microorganisms in monomicrobial BCs was 97%.

### 3.5. Antimicrobial Susceptibility for Pheno

Antimicrobial susceptibility results for Pheno were produced for 52 isolates with a total of 483 antimicrobial test results (38 Gram-positives and 445 Gram-negatives).  Table 4(a) lists the summary of Pheno discrepancy testing results for Gram-positives. Among them, 36 out of the 38 tests performed were concordant with the routine SensiTitre; 2 MEs (5.9%) were found for ampicillin and vancomycin, with a CA of 94.7% and EA of 82.9%. For a single* S. aureus* isolate, Pheno failed the MIC calculation of the cefoxitin screening test and did not provide the result. The AST results of Gram-negatives, the total percent errors as well as the percent error for each antimicrobial agent are shown in Table 4(b). Among Gram-negatives, 4 VMEs (3.0%) involved piperacillin/tazobactam (*n* = 2), ertapenem (*n* = 1), and meropenem (*n* = 1). For 3 carbapenemase-producing* Klebsiella pneumoniae* isolates, the meropenem MIC value was below the breakpoint; nevertheless, we considered the result as concordant, since Pheno provided a note advising for the presence of carbapenemase-producer microorganism. Thirteen MEs (4.6%) were referred to ampicillin/sulbactam (*n* = 3), piperacillin/tazobactam (*n* = 3), gentamicin (*n* = 3), colistin (*n* = 2), cefepime (*n* = 1), and ceftazidime (*n* = 1). There were 26 MiEs (5.84%) for cefepime (*n* = 6), meropenem (*n* = 5), amikacin (*n* = 5), piperacillin/tazobactam (*n* = 4), ceftazidime (*n* = 2), ciprofloxacin (*n* = 2), ertapenem (*n* = 1), and gentamicin (*n* = 1). Since with Alfred 60AST polymicrobial cultures could not be processed, we decided to remove them from Pheno analysis also. Excluding the polymicrobial BCs, 428 tests for Gram-negatives and 34 tests for Gram-positives were analyzed. The CA and EA of Gram-positives were 100% and 87%, respectively, while for Gram-negatives the CA was 90.6% (including 4 VMEs (3.2%), 13 MEs (4.5%), and 23 MiEs (4.4%)) and the EA was 81.4%. These results show that polymicrobial cultures slightly affect the CA for Pheno, mostly for Gram-positive bacteria. Further studies, mainly focalized on polymicrobial BCs, might be necessary in order to assess the performances of Pheno on this type of samples, as also reported elsewhere [29]. The overall categorical agreement of the 496 AST results was 91.3% with minor, major, and very major errors occurring at a rate of 5.0, 4.1, and 3.1%, respectively.

### 3.6. Antimicrobial Susceptibility for Alfred 60AST

**Figure 2:**
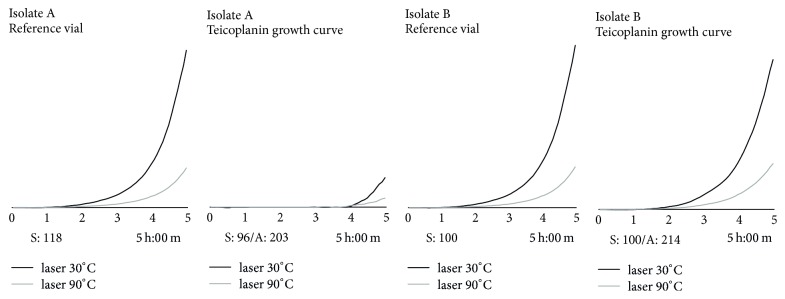
Isolate A represents an example of a false teicoplanin-resistant strain: in the presence of the drug, the microorganism seems to start growing in the last 30 minutes of the analysis. Isolate B represents a true teicoplanin-resistant strain: the growth curve in the presence of the drug is comparable to the reference vial. The horizontal bar under the figures represents incubation time (5 hours). Laser 30°C and laser 90°C represent the two detectors of the instrument. S sample; A antibiotics.

Antimicrobial susceptibility results for Alfred 60AST were produced for 58 isolates with a total of 405 antimicrobial test results (47 Gram-positives and 358 Gram-negatives). The time needed to reach a bacterial density corresponding to 0.5 McFarland ranged between 1-2 hours for Gram-negatives (median time 1 h and 40 min, ±18 min) and 1–3 hours for Gram-positives (median time 1 h and 55 min, ±39 min), depending on bacterial species. For Gram-positive bacteria, the CA was 85.1%. Seven MEs (17.1%) were found for linezolid (*n* = 1), vancomycin (*n* = 2), and teicoplanin (*n* = 4). For glycopeptides, we experienced several tests with MEs [27, 30]; however, with a careful observation of growth curves, it was possible to realize that the interpretation was incorrect (Figure 2). Hence, with a manual correction of ambiguous growth curves, the agreement for Gram-positives was of 95.7%, with 2 MEs only (4.9%).  Table 5(a) lists the summary of Alfred 60AST discrepancy testing results for Gram-positives. As reported in Table 5(b) the CA for Gram-negatives was 91.1%. Sixteen VMEs (15.4%) involved levofloxacin (*n* = 5), gentamicin (*n* = 4), colistin (*n* = 2), ceftazidime (*n* = 2), cefotaxime (*n* = 1), and trimethoprim-sulfamethoxazole (*n* = 2). There were 11 MEs (4.4%) involving trimethoprim-sulfamethoxazole (*n* = 1), gentamicin (*n* = 1), meropenem (*n* = 2), and piperacillin/tazobactam (*n* = 7). Finally, 5 MiEs (1.4%) were detected among ceftazidime (*n* = 2) and piperacillin/tazobactam (*n* = 3). The overall CA of the 405 AST results was of 91.6% with minor, major, and very major errors occurring at a rate of 1.2, 4.5, and 14.5%, respectively.

### 3.7. Direct Comparison of Alfred 60AST versus Pheno

Direct comparison between the two methodologies was possible for 261 AST results. Two hundred and twenty-five tests (86.2%) were concordant: among these, 85.4% for Gram-negatives and 95.2% for Gram-positives. Five concordant tests (1.9%) were discordant with SensiTitre, particularly gentamicin (*n* = 1), ceftazidime (*n* = 1), and piperacillin/tazobactam (*n* = 3). In the latter cases, we used the MIC test strip and the SensiTitre results were confirmed. These results suggest that the majority of issues for both methods were represented by piperacillin/tazobactam, which is known to be unstable [31]. Details of AST results for each antibiotic are presented in Table 6.

## 4. Conclusions

Regardless of its precise definition, sepsis is recorded as the most expensive condition in hospitals all over the world [1, 3, 32]. Early appropriate antimicrobial regimen, especially in the case of multidrug resistant bacteria, is pivotal to decrease mortality and may contribute to reducing healthcare costs [33, 34]. An additional predictable benefit is a reduction in the escalating rate of resistances, estimated to cause 10 million deaths per year worldwide in the next 30 years, with a cumulative economic cost of US$100 trillion [32]. The optimal duration of therapy for a bloodstream infection would be long enough to effectively eradicate infection and prevent relapse, while short enough to limit adverse effects, avoid secondary infections by opportunistic pathogens, and minimize selective pressure for antibiotic resistance [4]. In our hospital, given the high prevalence of multidrug resistant pathogens, there is an urgent need of rapid antimicrobial susceptibility test results during sepsis. For this reason, we evaluated the performance of the Pheno system and MALDI-TOF MS coupled with Alfred 60AST system for rapid identification and antimicrobial susceptibility tests of microorganisms directly from positive blood cultures. To our knowledge, this is the first evaluation of the two methodologies in comparison to the SensiTitre broth microdilution assay, both on Gram-positive and Gram-negative bacteria.

The Pheno system performed well for the identification of bloodstream pathogens, correctly identifying 97% of microorganisms. These results are in agreement with a recent study conducted only on Gram-negative bacteria [29]. It is worthy of notice that the system failed the growth control for various reasons for 12% monomicrobial BCs and, since Pheno is a closed system, there is no possibility for the operator to intervene and fix the eventual inconvenience. The failed tests are slightly lower than the proportion reported elsewhere [35]; nevertheless, the studies about this aspect are still partial. Pheno presents the limitation of processing only fresh BCs (within 8 hours after a BC turns positive). Furthermore, for many microorganisms, only the* genus* was available; however, identification to the species level may be important for infection control purposes and epidemiological studies. In addition, Pheno detected a second organism in 2 monomicrobial BCs according to the routine culture method, precisely* Serratia marcescens* in a BC positive only for* Enterobacter *spp., and CoNS coupled with an* Enterococcus faecium* strain. Performance on AST data was good both for Gram-positive and Gram-negative microorganisms, with an overall categorical agreement of 100% and 90.6%, respectively. There were 4 false-susceptible and 13 false-resistant results, affecting mainly *β*-lactams, gentamicin and colistin. The categorical and essential agreements obtained in the present study were slightly lower (CA 91.3% versus 95.5% and EA 81.8% versus 95.1%) than data available in a recently published article by Charnot-Katsikas and colleagues [35]. A possible explanation is that they used Vitek2 (bioMérieux) as a comparator, a system which operates on fewer dilutions and so may be less precise than the reference method used in the present study. A key point of Pheno is that the instrument is user-friendly and does not require skillful technicians or a well-trained staff. In addition, the time required to obtain a complete report including both the ID and AST was rapid: 3–5 min for cartridge set up, 80–120 min for ID, and about 5-6 hours for AST.

The Alfred 60AST system was coupled with MALDI-TOF MS for direct identification and AST in positive blood cultures. MALDI-TOF MS performances were optimal, since a correct identification was achieved in all the monomicrobial BCs analyzed. An interesting meta-analysis about direct MALDI-TOF ID from positive blood cultures suggested that MALDI-TOF provides highly accurate identification of Gram-negative bacteria at the species level, while for Gram-positive bacteria overall accuracy is moderate [36]. On the contrary, we obtained optimal results also with Gram-positives, probably because a valid protein extraction protocol was used. Regarding AST, Alfred 60AST performances were good, with an overall categorical agreement of 91.0% and 95.7% for Gram-negative and Gram-positive bacteria, respectively. The majority of problems involved the glycopeptides, particularly teicoplanin (Alifax). According to some experiments performed in our laboratory, glycopeptides (Alifax) lose their effectiveness when close to the expiration date, probably because the drug powder deteriorated over time. For this reason, the cap on the vial containing the lyophilized antibiotic was replaced by the company with a new version capable of guaranteeing greater resistance to external agents (e.g., humidity in the refrigerator cells), preserving the effectiveness of the drug. There were 16 false-susceptible and 18 false-resistant results, affecting mainly *β*-lactams, gentamicin, levofloxacin, and glycopeptides. Further, 2 VMEs affecting colistin were due to the duration of the test. Apparently, 3 hours were not enough to allow the resistance to be revealed by the algorithm; thus, incubation time for the colistin test was extended up to 5 hours. One of the main aspects of Alfred 60AST is the plasticity of the system, since there is the possibility of eventually intervening in the course of the AST. On the other hand, a trained staff, able to interpret the results and particularly the microbial growth curves, is required. Notably, the microbiology laboratory can decide the antibiotic to insert in the AST panel, according to patient needs. The time required to obtain a complete result including both the ID and AST was about 6 hours: 3–5 min for setting up vials, 15–20 min for MALDI-TOF MS ID, and about 4–6 hours for AST, depending on the chosen antibiotics.

In conclusion, both methodologies provided comparable results, showing no statistically significant differences. AST preliminary results were reliable for both and useful to start a proper antibiotic treatment, which can be confirmed afterwards with the traditional AST. The time to obtain ID and AST as well as costs are lower for Alfred 60AST combined with MALDI-TOF MS. On the other hand, Pheno provides both identification and MIC determination in one cartridge. Although certainly more expensive, Pheno can be useful in medium and small laboratories and when MIC values are necessary for an appropriate therapy. Both systems allow us to establish a proper diagnostic stewardship in order to hinder sepsis and minimize the spread of bacterial resistance.

## Figures and Tables

**(a):** 

Gram positives	Ampicillin	Ceftaroline	Doxycycline	Erythromycin	Trimethoprim-sulfamethoxazole	Daptomycin	Linezolid	Vancomycin	Cefoxitin	MLSb (erythromycin-clindamycin)
*S. aureus*		X	X	X	X	X	X	X	X	
*CoNS*			X	X		X	X	X	X	X
*S. lugdunensis*			X	X		X	X	X	X	X
*E. faecalis*	X					X	X	X		
*E. faecium*	X					X	X	X		
*Streptococcus spp.*										
*S. pneumoniae*										
*S. agalactiae*										

**(b):** 

Gram negatives	Ampicillin-sulbactam	Piperacillin tazobactam	Cefazolin	Cefepime	Ceftazidime	Ceftriaxone	Ertapenem	Meropenem	Amikacin	Gentamicin	Tobramycin	Ciprofloxacin	Minocycline	Aztreonam	Colistin
*E. coli*	X	X	X	X	X	X	X	X	X	X	X	X		X	X
*Klebsiella* spp.		X	X	X	X	X	X	X	X	X	X	X		X	X
*Enterobacter* spp.		X		X	X	X	X	X	X	X	X	X		X	X
*Proteus *spp.		X		X	X	X	X	X	X	X	X	X		X	
*Citrobacter *spp.		X		X	X	X	X	X	X	X	X	X		X	X
*S. marcescens*		X		X	X	X	X	X	X	X	X	X		X	
*A. baumannii*	X	X		X				X	X			X	X		X
*P. aeruginosa *		X		X	X			X	X	X	X	X		X	X

**Table 2:** Monomicrobial BCs: identification results of the direct MALDI-TOF MS ID and Pheno ID compared to culture-based ID.

Direct MALDI-TOF ID	MALDI-TOF score value				Accelerate Pheno ID	Accelerate Pheno comment	Total
	3.000–2.300	2.299–2.000	1.999–1.700	<1.700			
*Acinetobacter baumannii*		1			*Acinetobacter baumannii*		1

*Citrobacter koseri*	1	2			*Citrobacter *spp.		3

*Enterobacter cloacae*		2	2		*Enterobacter* spp. *Enterobacter* spp./*Serratia marcescens*	False identification, additional organism detected, no AST (*n* = 1); Gram-negative detected correctly, no AST, growth control failure (*n* = 1)	4

*Escherichia coli*	15	4			*Escherichia coli*	Gram-negative detected correctly, no AST, growth control failure (*n* = 1)	19

*Klebsiella pneumoniae*	13	6			*Klebsiella* spp.	Gram-negative detected correctly, no AST, growth control failure (*n* = 2)	19

*Proteus mirabilis*		1			*Proteus* spp.		1

*Pseudomonas aeruginosa*	1				*Pseudomonas aeruginosa*		1

*Serratia marcescens*		2			*Serratia marcescens*		2

*Enterococcus faecalis*		1			*Enterococcus faecalis*		1

*Enterococcus faecium*	1	2			*Enterococcus faecium* *Enterococcus faecium*/CoNS	False identification, additional organism detected, no AST (*n* = 1)	3

*Staphylococcus aureus*	1	2	1		*Staphylococcus aureus*	Gram-positive detected correctly, no AST, growth control failure (*n* = 1)	4

*Streptococcus agalactiae*	1				*Streptococcus agalactiae*	Organism ineligible for susceptibility testing (*n* = 1)	1

*Streptococcus pneumoniae*		1			*Streptococcus* spp.	Organism ineligible for susceptibility testing (*n* = 1)	1

Total	33	24	3				60

**Table 3:** Polymicrobial BCs: identification results of the direct MALDI-TOF MS ID and Pheno ID compared to culture-based ID.

Direct MALDI-TOF ID	MALDI-TOF score value				Additional organism from culture ID	Accelerate Pheno ID	Comment
	3.000–2.300	2.299–2.000	1.999–1.700	<1.700			
*Enterobacter cloacae*		1			*Staphylococcus warneri*	No identification	Suspected off-panel, no AST
*Klebsiella oxytoca*	1				*Citrobacter freundi*	*Klebsiella* spp.	*Klebsiella* spp. detected correctly
*Klebsiella pneumoniae*		1			*Staphylococcus haemolyticus*	*Klebsiella* spp.	Gram-negative organism detected correctly
*Klebsiella pneumoniae*		1			*Bacillus circulans*		*Klebsiella* spp./additional bacteria or yeasts, no AST, growth control failure
*Enterococcus faecalis*		1			*Pseudomonas oryzihabitans*	*Enterococcus faecalis*	Gram-positive organism detected correctly, no AST
*Enterococcus faecalis*	1				*Streptococcus parasanguinis*	*Enterococcus faecalis*	Gram-positive organism detected correctly, no AST
*Enterococcus faecalis*		1			*Klebsiella pneumoniae*	*Enterococcus faecalis*, *Klebsiella* spp.	Gram-negative organism detected correctly, no AST

**(a):** 

Antimicrobial agent	Number of very major errors (%)	Number of major errors (%)	Number of minor errors (%)	AST agreement (%)	Total
Gram-positives					
Ampicillin	0/2	1/2 (50.0)	0/4	3/4 (75.0)	4
Ceftaroline	0/0	0/3	0/3	3/3 (100.0)	3
Doxycycline	0/0	0/2	0/2	2/2 (100.0)	2
Erythromycin	0/1	0/2	0/3	3/3 (100.0)	3
Trimethoprim-sulfamethoxazole	0/0	0/3	0/3	3/3 (100.0)	3
Daptomycin	0/0	0/5	0/5	5/5 (100.0)	5
Linezolid	0/0	0/7	0/7	7/7 (100.0)	7
Vancomycin	0/1	1/6 (16.7)	0/7	6/7 (85.7)	7
Cefoxitin	0/0	0/2	0/2	2/2 (100.0)	2
MLS screening	0/0	0/2	0/2	2/2 (100.0)	2
Total	0/4	2/34 (5.9)	0/38	36/38 (94.7)	38

**(b):** 

Antimicrobial agent	Number of very major errors (%)	Number of major errors (%)	Number of minor errors (%)	AST agreement (%)	Total
Gram-negatives					
Ampicillin/sulbactam	0/27	3/7 (42.9)	0/34	31/34 (91.2)	34
Piperacillin/tazobactam	2/15 (13.3)	3/28 (10.7)	4/46 (8.7)	37/46 (80.4)	46
Cefazoline	0/1	0/0	0/1	1/1 (100.0)	1
Cefepime	0/13	1/30 (3.3)	6/46 (13.0)	39/46 (84.8)	46
Ceftazidime	0/15	1/28 (3.6)	2/45 (4.4)	42/45 (93.3)	45
Cefotaxime^*∗*^	0/0	0/1	0/1	1/1 (100.0)	1
Ertapenem	1/9 (11.1)	0/35	1/44 (2.3)	42/44 (95.5)	44
Meropenem	1/9 (11.1)	0/37	5/46 (10.9)	40/46 (87.0)	46
Gentamicin	0/7	3/38 (7.9)	1/45 (2.2)	41/45 (91.1)	45
Amikacin	0/9	0/33	5/46 (10.9)	41/46 (89.1)	46
Tobramycin	0/0	0/2	0/2	2/2 (100.0)	2
Ciprofloxacin	0/22	0/22	2/46 (4.4)	44/46 (95.7)	46
Colistin	0/5	2/38 (5.3)	0/43	41/43 (95.4)	43
Total	4/132 (3.0)	13/299 (4.4)	26/445 (5.8)	402/445 (90.3)	445

**(a):** 

Antimicrobial agent	Number of very major errors (%)	Number of major errors (%)	Number of minor errors (%)	AST agreement (%)	Total
Gram-positives					
Ampicillin	0/3	0/2	0/5	5/5 (100.0)	5
Daptomycin	0/0	0/8	0/8	8/8 (100.0)	8
Linezolid	0/0	1/10 (10.0)	0/10	9/10 (90.0)	10
Vancomycin	0/1	2/6 (33.3)	0/7	5/7 (71.4)	7
Teicoplanin	0/1	4/8 (50.0)	0/9	5/9 (55.6)	9
Cefoxitin	0/1	0/3	0/4	4/4 (100.0)	4
Clindamycin	0/0	0/4	0/4	4/4 (100.0)	4
Total	0/6	7/41 (17.1)	0/47	40/47 (85.1)	47

**(b):** 

Antimicrobial agent	Number of very major errors (%)	Number of major errors (%)	Number of minor errors (%)	AST agreement (%)	Total
Gram-negatives					
Piperacillin/tazobactam	0/15	7/26 (26.9)	3/44 (6.8)	34/44 (77.3)	44
Ceftazidime	2/14 (14.3)	0/30	2/46 (4.4)	42/46 (91.3)	46
Cefotaxime	1/14 (7.1)	0/31	0/45	44/45 (97.8)	45
Meropenem	0/8	2/38 (5.3)	0/46	44/46 (95.7)	46
Gentamicin	4/8 (50.0)	1/37 (2.7)	0/45	40/45 (88.9)	45
Colistin	2/6 (33.3)	0/37	0/43	41/43 (95.4)	43
Levofloxacin	5/20 (25.0)	0/24	0/44	39/44 (88.6)	44
Trimethoprim-sulfamethoxazole	2/18 (11.1)	1/23 (4.4)	0/41	38/41 (92.7)	41
Cefuroxime	0/1	0/3	0/4	4/4 (100.0)	
Total	16/104 (15.4)	11/249 (4.4)	5/358 (1.4)	326/358 (91.1)	358

**(a):** 

Antimicrobial agent	Number of discrepancies (%)	AST agreement (%)	Total
Gram-positives			
Ampicillin	0/3	3/3 (100.0)	3
Daptomycin	0/4	4/4 (100.0)	4
Linezolid	0/6	6/6 (100.0)	6
Vancomycin	1/5 (20.0)	4/5 (80.0)	5
Cefoxitin	0/2	2/2 (100.0)	2
Clindamycin	0/1	1/1 (100.0)	1
Total	1/21 (4.8)	20/21 (95.2)	21

**(b):** 

Antimicrobial agent	Number of discrepancies (%)	AST agreement (%)	Total
Gram-negatives			
Piperacillin/tazobactam	11/40 (27.5)	29/40 (72.5)	40
Ceftazidime	4/41 (9.8)	37/41 (90.2)	41
Ceftriaxone	3/39 (7.7)	36/39 (92.3)	39
Cefotaxime	0/1	1/1 (100.0)	1
Meropenem	7/41 (17.1)	34/41 (82.9)	41
Gentamicin	6/40 (15.0)	34/40 (85.0)	40
Colistin	4/38 (10.5)	34/38 (89.5)	38
Total	35/240 (14.6)	205/240 (85.4)	240
